# 4006 Methionine Dependence in Cancer: From Metabolic Phenotype to Therapy

**DOI:** 10.1017/cts.2020.81

**Published:** 2020-07-29

**Authors:** Isabelle Miousse

**Affiliations:** 1 University of Arkansas Translational Research Institute

## Abstract

OBJECTIVES/GOALS: Methionine dependence was described 45 years ago as an increased reliance on an exogenous supply of the essential amino acid methionine in most cancer cells compared to normal cells. Methionine depletion, using either synthetic diets or the enzyme methioninase, potentiates the effects of chemotherapy and radiotherapy in tumor-bearing animal models. Two main obstacles prevent methionine dependence from integrating the clinical treatment of cancer. The first is the weight loss associated with methionine depletion therapy, increasing the risk of cachexia in patients. The second is the stubborn absence of a mechanism to explain the inability of cancer cells to adapt to low methionine levels. METHODS/STUDY POPULATION: To address these two obstacles, we are using an immunocompetent murine model of metastatic melanoma to compare the effects of complete methionine deprivation with a moderate, 75-80% methionine restriction similar to the one used to increase lifespan in animal models. In an effort to identify a mechanism of action, we also performed a proteomic screen of two melanoma cell lines divergent for methionine dependence under methionine stress. RESULTS/ANTICIPATED RESULTS: We recently showed that methionine restriction is sufficient to provide gains in treating local and metastatic lesions in vivo, without weight loss. We observed few differences in pathway activation between the two cell lines in response to methionine stress, despite proliferation being cut by half in the methionine dependent cell line. We expect that subcellular translocation events may provide further information on the molecular bases of methionine dependence. DISCUSSION/SIGNIFICANCE OF IMPACT: A moderate restriction in methionine is sufficient to recapitulate the benefits of methionine depletion in cancer, without weight loss. The mechanism behind this effect remains unknown. This work contributes towards the integration of methionine dependence into clinical practice and the discovery of novel drug targets.

